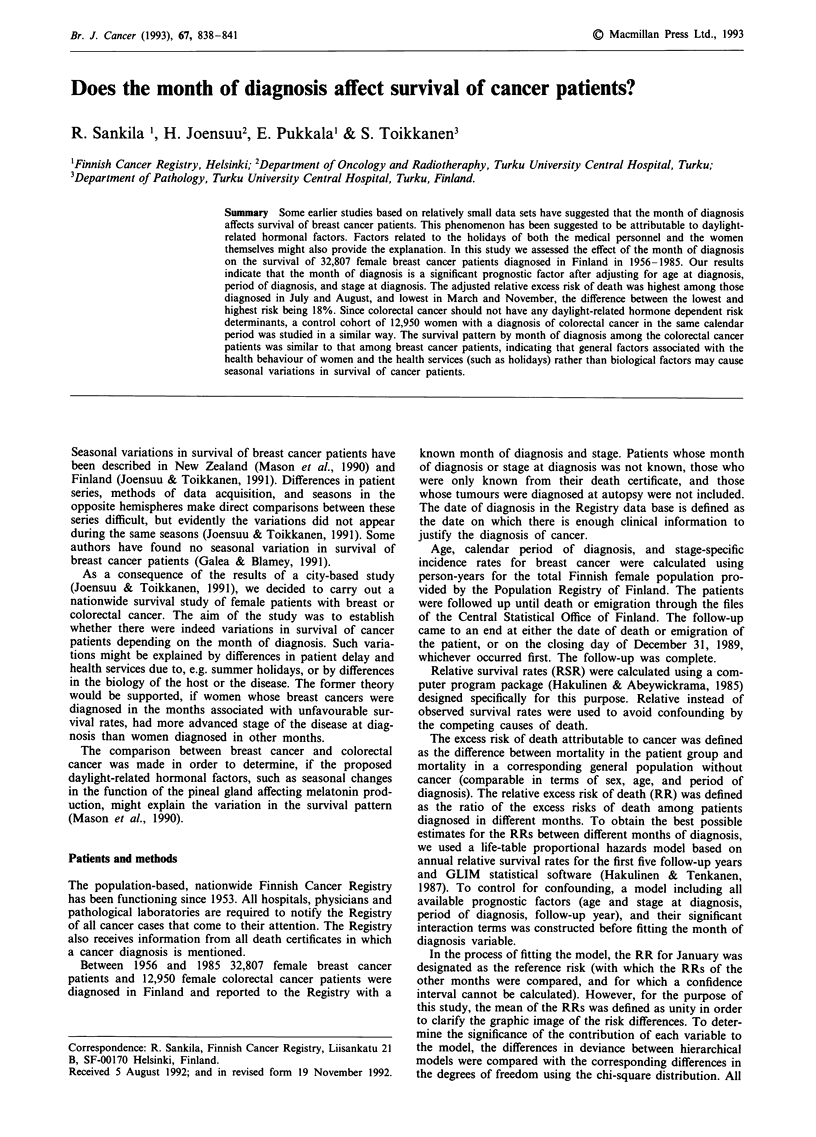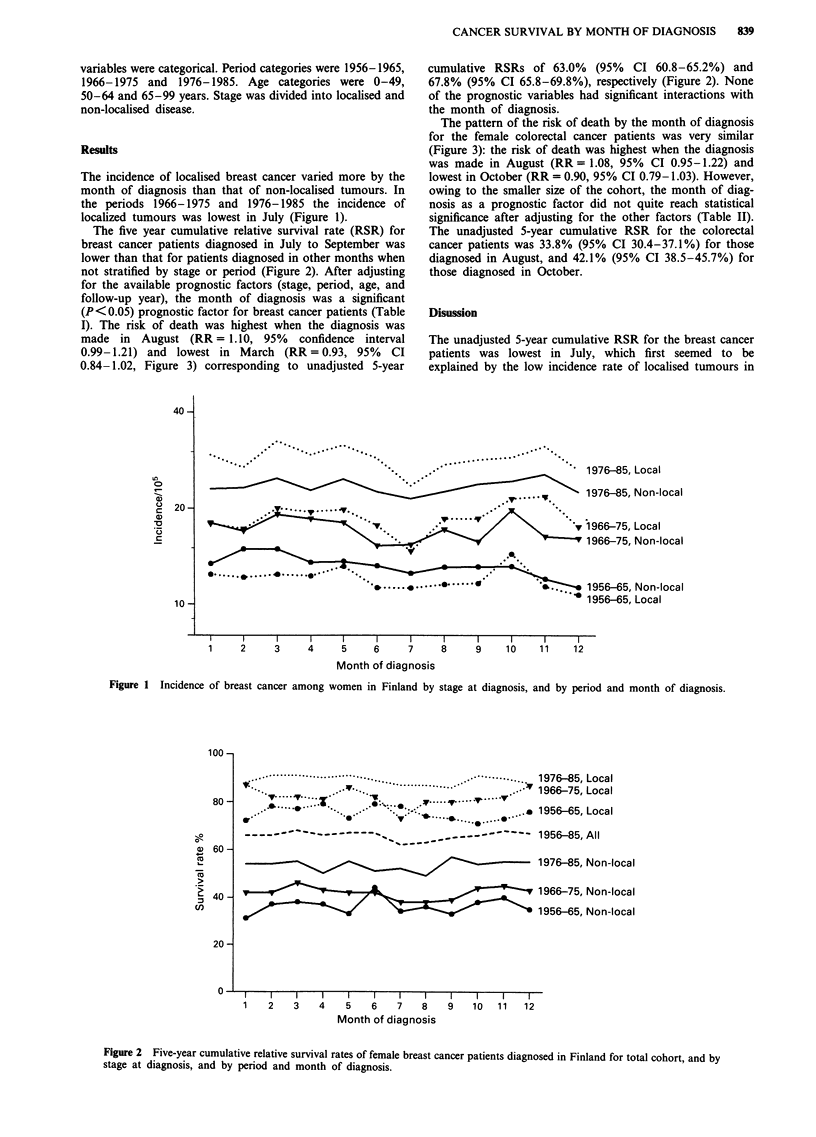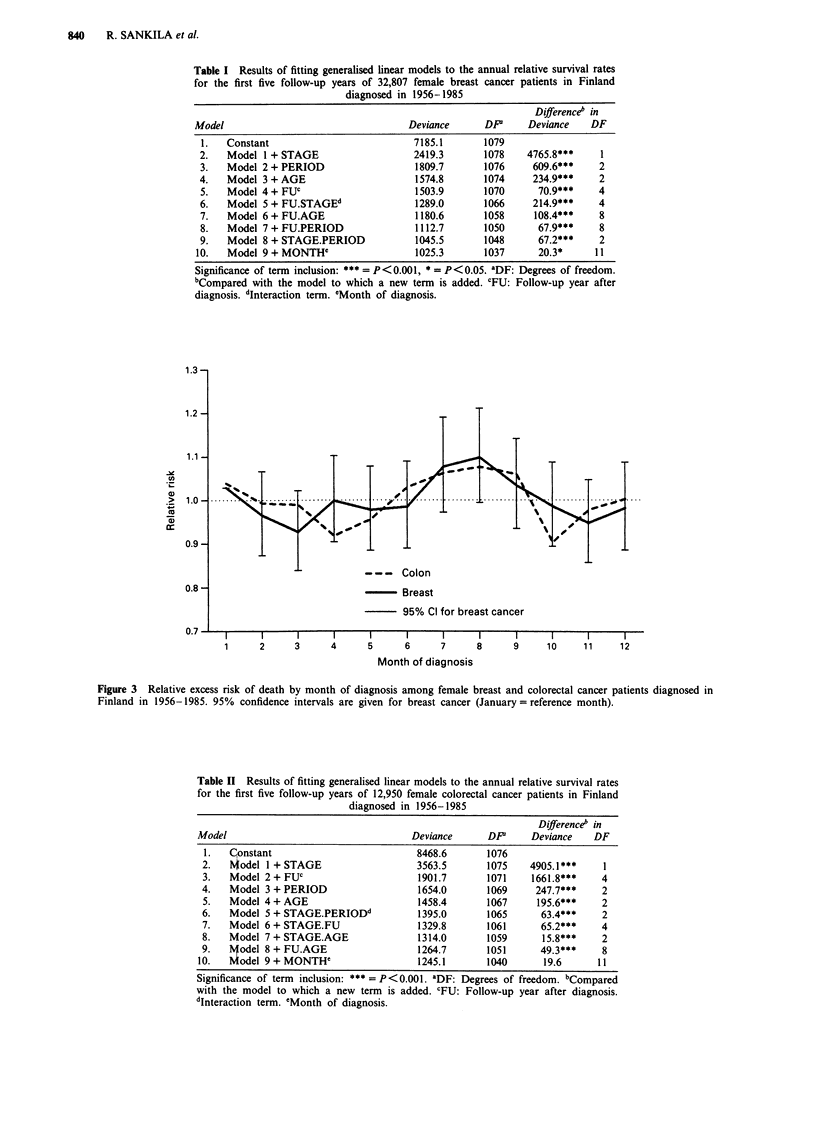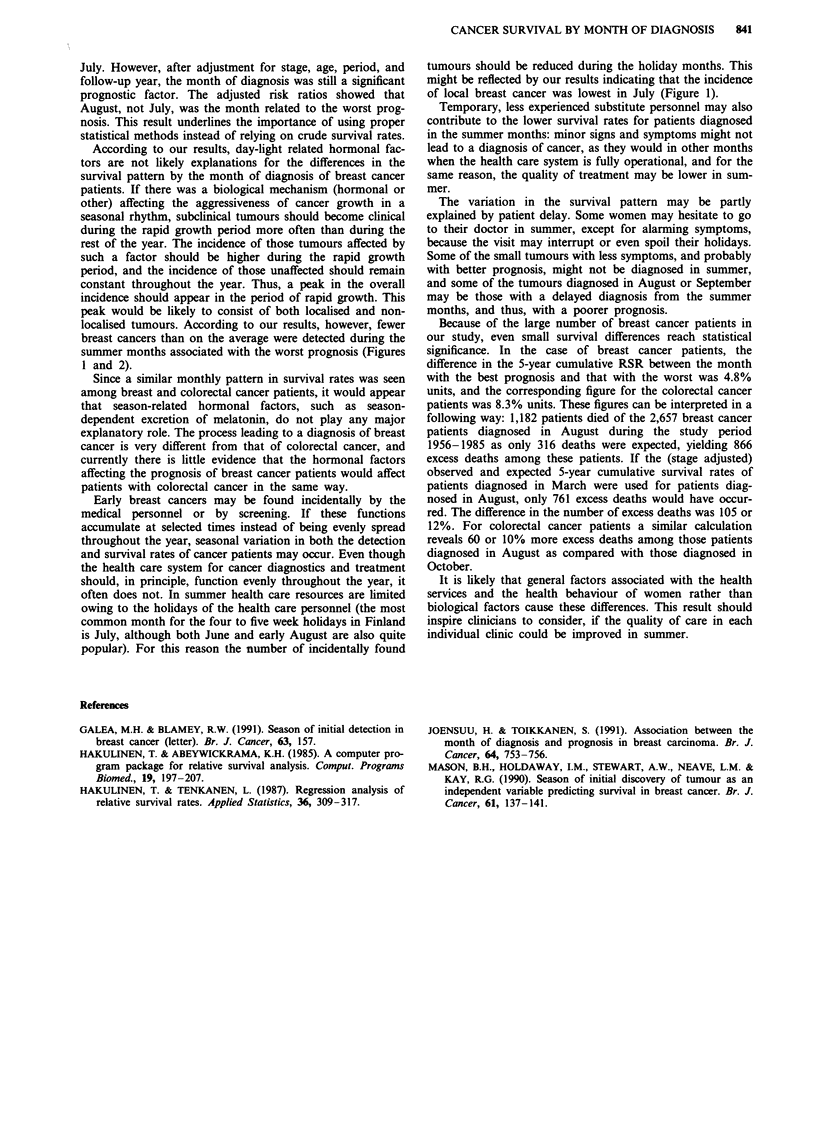# Does the month of diagnosis affect survival of cancer patients?

**DOI:** 10.1038/bjc.1993.153

**Published:** 1993-04

**Authors:** R. Sankila, H. Joensuu, E. Pukkala, S. Toikkanen

**Affiliations:** Finnish Cancer Registry, Helsinki.

## Abstract

Some earlier studies based on relatively small data sets have suggested that the month of diagnosis affects survival of breast cancer patients. This phenomenon has been suggested to be attributable to daylight-related hormonal factors. Factors related to the holidays of both the medical personnel and the women themselves might also provide the explanation. In this study we assessed the effect of the month of diagnosis on the survival of 32,807 female breast cancer patients diagnosed in Finland in 1956-1985. Our results indicate that the month of diagnosis is a significant prognostic factor after adjusting for age at diagnosis, period of diagnosis, and stage at diagnosis. The adjusted relative excess risk of death was highest among those diagnosed in July and August, and lowest in March and November, the difference between the lowest and highest risk being 18%. Since colorectal cancer should not have any daylight-related hormone dependent risk determinants, a control cohort of 12,950 women with a diagnosis of colorectal cancer in the same calendar period was studied in a similar way. The survival pattern by month of diagnosis among the colorectal cancer patients was similar to that among breast cancer patients, indicating that general factors associated with the health behaviour of women and the health services (such as holidays) rather than biological factors may cause seasonal variations in survival of cancer patients.


Br. J. Cancer (1993), 67, 838–841 © Macmillan Press Ltd., 1993

### Does the month of diagnosis affect survival of cancer patients?

R. Sankila [1], H. Joensuu[2], E. Pukkala[1] & S. Toikkanen[3]

[1]*Finnish Cancer Registry, Helsinki;* [2]*Department of Oncology and Radiotheraphy, Turku University Central Hospital, Turku;* [3]*Department of Pathology, Turku University Central Hospital, Turku, Finland.*

**Summary** Some earlier studies based on relatively small data sets have suggested that the month of diagnosis affects survival of breast cancer patients. This phenomenon has been suggested to be attributable to daylight-related hormonal factors. Factors related to the holidays of both the medical personnel and the women themselves might also provide the explanation. In this study we assessed the effect of the month of diagnosis on the survival of 32,807 female breast cancer patients diagnosed in Finland in 1956–1985. Our results indicate that the month of diagnosis is a significant prognostic factor after adjusting for age at diagnosis, period of diagnosis, and stage at diagnosis. The adjusted relative excess risk of death was highest among those diagnosed in July and August, and lowest in March and November, the difference between the lowest and highest risk being 18%. Since colorectal cancer should not have any daylight-related hormone dependent risk determinants, a control cohort of 12,950 women with a diagnosis of colorectal cancer in the same calendar period was studied in a similar way. The survival pattern by month of diagnosis among the colorectal cancer patients was similar to that among breast cancer patients, indicating that general factors associated with the health behaviour of women and the health services (such as holidays) rather than biological factors may cause seasonal variations in survival of cancer patients.

Seasonal variations in survival of breast cancer patients have been described in New Zealand (Mason *et al.*, 1990) and Finland (Joensuu & Toikkanen, 1991). Differences in patient series, methods of data acquisition, and seasons in the opposite hemispheres make direct comparisons between these series difficult, but evidently the variations did not appear during the same seasons (Joensuu & Toikkanen, 1991). Some authors have found no seasonal variation in survival of breast cancer patients (Galea & Blamey, 1991).

As a consequence of the results of a city-based study (Joensuu & Toikkanen, 1991), we decided to carry out a nationwide survival study of female patients with breast or colorectal cancer. The aim of the study was to establish whether there were indeed variations in survival of cancer patients depending on the month of diagnosis. Such variations might be explained by differences in patient delay and health services due to, e.g. summer holidays, or by differences in the biology of the host or the disease. The former theory would be supported, if women whose breast cancers were diagnosed in the months associated with unfavourable survival rates, had more advanced stage of the disease at diagnosis than women diagnosed in other months.

The comparison between breast cancer and colorectal cancer was made in order to determine, if the proposed daylight-related hormonal factors, such as seasonal changes in the function of the pineal gland affecting melatonin production, might explain the variation in the survival pattern (Mason *et al.*, 1990).

### Patients and methods

The population-based, nationwide Finnish Cancer Registry has been functioning since 1953. All hospitals, physicians and pathological laboratories are required to notify the Registry of all cancer cases that come to their attention. The Registry also receives information from all death certificates in which a cancer diagnosis is mentioned.

Between 1956 and 1985 32,807 female breast cancer patients and 12,950 female colorectal cancer patients were diagnosed in Finland and reported to the Registry with a known month of diagnosis and stage. Patients whose month of diagnosis or stage at diagnosis was not known, those who were only known from their death certificate, and those whose tumours were diagnosed at autopsy were not included. The date of diagnosis in the Registry data base is defined as the date on which there is enough clinical information to justify the diagnosis of cancer.

Age, calendar period of diagnosis, and stage-specific incidence rates for breast cancer were calculated using person-years for the total Finnish female population provided by the Population Registry of Finland. The patients were followed up until death or emigration through the files of the Central Statistical Office of Finland. The follow-up came to an end at either the date of death or emigration of the patient, or on the closing day of December 31, 1989, whichever occurred first. The follow-up was complete.

Relative survival rates (RSR) were calculated using a computer program package (Hakulinen & Abeywickrama, 1985) designed specifically for this purpose. Relative instead of observed survival rates were used to avoid confounding by the competing causes of death.

The excess risk of death attributable to cancer was defined as the difference between mortality in the patient group and mortality in a corresponding general population without cancer (comparable in terms of sex, age, and period of diagnosis). The relative excess risk of death (RR) was defined as the ratio of the excess risks of death among patients diagnosed in different months. To obtain the best possible estimates for the RRs between different months of diagnosis, we used a life-table proportional hazards model based on annual relative survival rates for the first five follow-up years and GLIM statistical software (Hakulinen & Tenkanen, 1987). To control for confounding, a model including all available prognostic factors (age and stage at diagnosis, period of diagnosis, follow-up year), and their significant interaction terms was constructed before fitting the month of diagnosis variable.

In the process of fitting the model, the RR for January was designated as the reference risk (with which the RRs of the other months were compared, and for which a confidence interval cannot be calculated). However, for the purpose of this study, the mean of the RRs was defined as unity in order to clarify the graphic image of the risk differences. To determine the significance of the contribution of each variable to the model, the differences in deviance between hierarchical models were compared with the corresponding differences in the degrees of freedom using the chi-square distribution. All

Correspondence: R. Sankila, Finnish Cancer Registry, Liisankatu 21 B, SF-00170 Helsinki, Finland.
Received 5 August 1992; and in revised form 19 November 1992.

variables were categorical. Period categories were 1956–1965, 1966–1975 and 1976–1985. Age categories were 0–49, 50–64 and 65–99 years. Stage was divided into localised and non-localised disease.

### Results

The incidence of localised breast cancer varied more by the month of diagnosis than that of non-localised tumours. In the periods 1966–1975 and 1976–1985 the incidence of localized tumours was lowest in July (Figure 1).

The five year cumulative relative survival rate (RSR) for breast cancer patients diagnosed in July to September was lower than that for patients diagnosed in other months when not stratified by stage or period (Figure 2). After adjusting for the available prognostic factors (stage, period, age, and follow-up year), the month of diagnosis was a significant ($P<0.05$) prognostic factor for breast cancer patients (Table I). The risk of death was highest when the diagnosis was made in August (RR = 1.10, 95% confidence interval 0.99–1.21) and lowest in March (RR = 0.93, 95% CI 0.84–1.02, Figure 3) corresponding to unadjusted 5-year cumulative RSRs of 63.0% (95% CI 60.8–65.2%) and 67.8% (95% CI 65.8–69.8%), respectively (Figure 2). None of the prognostic variables had significant interactions with the month of diagnosis.

The pattern of the risk of death by the month of diagnosis for the female colorectal cancer patients was very similar (Figure 3): the risk of death was highest when the diagnosis was made in August (RR = 1.08, 95% CI 0.95–1.22) and lowest in October (RR = 0.90, 95% CI 0.79–1.03). However, owing to the smaller size of the cohort, the month of diagnosis as a prognostic factor did not quite reach statistical significance after adjusting for the other factors (Table II). The unadjusted 5-year cumulative RSR for the colorectal cancer patients was 33.8% (95% CI 30.4–37.1%) for those diagnosed in August, and 42.1% (95% CI 38.5–45.7%) for those diagnosed in October.

### Disussion

The unadjusted 5-year cumulative RSR for the breast cancer patients was lowest in July, which first seemed to be explained by the low incidence rate of localised tumours in

**Figure 1** Incidence of breast cancer among women in Finland by stage at diagnosis, and by period and month of diagnosis.

**Figure 2** Five-year cumulative relative survival rates of female breast cancer patients diagnosed in Finland for total cohort, and by stage at diagnosis, and by period and month of diagnosis.

**Table I** Results of fitting generalised linear models to the annual relative survival rates for the first five follow-up years of 32,807 female breast cancer patients in Finland diagnosed in 1956–1985

| Model | | Deviance | DF[a] | Difference[b] in Deviance | Difference[b] in DF |
|---|---|---|---|---|---|
| 1. | Constant | 7185.1 | 1079 | | |
| 2. | Model 1 + STAGE | 2419.3 | 1078 | 4765.8*** | 1 |
| 3. | Model 2 + PERIOD | 1809.7 | 1076 | 609.6*** | 2 |
| 4. | Model 3 + AGE | 1574.8 | 1074 | 234.9*** | 2 |
| 5. | Model 4 + FU[c] | 1503.9 | 1070 | 70.9*** | 4 |
| 6. | Model 5 + FU.STAGE[d] | 1289.0 | 1066 | 214.9*** | 4 |
| 7. | Model 6 + FU.AGE | 1180.6 | 1058 | 108.4*** | 8 |
| 8. | Model 7 + FU.PERIOD | 1112.7 | 1050 | 67.9*** | 8 |
| 9. | Model 8 + STAGE.PERIOD | 1045.5 | 1048 | 67.2*** | 2 |
| 10. | Model 9 + MONTH[e] | 1025.3 | 1037 | 20.3* | 11 |

Significance of term inclusion: *** = $P < 0.001$, * = $P < 0.05$. [a]DF: Degrees of freedom. [b]Compared with the model to which a new term is added. [c]FU: Follow-up year after diagnosis. [d]Interaction term. [e]Month of diagnosis.

**Figure 3** Relative excess risk of death by month of diagnosis among female breast and colorectal cancer patients diagnosed in Finland in 1956–1985. 95% confidence intervals are given for breast cancer (January = reference month).

**Table II** Results of fitting generalised linear models to the annual relative survival rates for the first five follow-up years of 12,950 female colorectal cancer patients in Finland diagnosed in 1956–1985

| Model | | Deviance | DF[a] | Difference[b] in Deviance | Difference[b] in DF |
|---|---|---|---|---|---|
| 1. | Constant | 8468.6 | 1076 | | |
| 2. | Model 1 + STAGE | 3563.5 | 1075 | 4905.1*** | 1 |
| 3. | Model 2 + FU[c] | 1901.7 | 1071 | 1661.8*** | 4 |
| 4. | Model 3 + PERIOD | 1654.0 | 1069 | 247.7*** | 2 |
| 5. | Model 4 + AGE | 1458.4 | 1067 | 195.6*** | 2 |
| 6. | Model 5 + STAGE.PERIOD[d] | 1395.0 | 1065 | 63.4*** | 2 |
| 7. | Model 6 + STAGE.FU | 1329.8 | 1061 | 65.2*** | 4 |
| 8. | Model 7 + STAGE.AGE | 1314.0 | 1059 | 15.8*** | 2 |
| 9. | Model 8 + FU.AGE | 1264.7 | 1051 | 49.3*** | 8 |
| 10. | Model 9 + MONTH[e] | 1245.1 | 1040 | 19.6 | 11 |

Significance of term inclusion: *** = $P < 0.001$. [a]DF: Degrees of freedom. [b]Compared with the model to which a new term is added. [c]FU: Follow-up year after diagnosis. [d]Interaction term. [e]Month of diagnosis.

July. However, after adjustment for stage, age, period, and follow-up year, the month of diagnosis was still a significant prognostic factor. The adjusted risk ratios showed that August, not July, was the month related to the worst prognosis. This result underlines the importance of using proper statistical methods instead of relying on crude survival rates.

According to our results, day-light related hormonal factors are not likely explanations for the differences in the survival pattern by the month of diagnosis of breast cancer patients. If there was a biological mechanism (hormonal or other) affecting the aggressiveness of cancer growth in a seasonal rhythm, subclinical tumours should become clinical during the rapid growth period more often than during the rest of the year. The incidence of those tumours affected by such a factor should be higher during the rapid growth period, and the incidence of those unaffected should remain constant throughout the year. Thus, a peak in the overall incidence should appear in the period of rapid growth. This peak would be likely to consist of both localised and non-localised tumours. According to our results, however, fewer breast cancers than on the average were detected during the summer months associated with the worst prognosis (Figures 1 and 2).

Since a similar monthly pattern in survival rates was seen among breast and colorectal cancer patients, it would appear that season-related hormonal factors, such as season-dependent excretion of melatonin, do not play any major explanatory role. The process leading to a diagnosis of breast cancer is very different from that of colorectal cancer, and currently there is little evidence that the hormonal factors affecting the prognosis of breast cancer patients would affect patients with colorectal cancer in the same way.

Early breast cancers may be found incidentally by the medical personnel or by screening. If these functions accumulate at selected times instead of being evenly spread throughout the year, seasonal variation in both the detection and survival rates of cancer patients may occur. Even though the health care system for cancer diagnostics and treatment should, in principle, function evenly throughout the year, it often does not. In summer health care resources are limited owing to the holidays of the health care personnel (the most common month for the four to five week holidays in Finland is July, although both June and early August are also quite popular). For this reason the number of incidentally found tumours should be reduced during the holiday months. This might be reflected by our results indicating that the incidence of local breast cancer was lowest in July (Figure 1).

Temporary, less experienced substitute personnel may also contribute to the lower survival rates for patients diagnosed in the summer months: minor signs and symptoms might not lead to a diagnosis of cancer, as they would in other months when the health care system is fully operational, and for the same reason, the quality of treatment may be lower in summer.

The variation in the survival pattern may be partly explained by patient delay. Some women may hesitate to go to their doctor in summer, except for alarming symptoms, because the visit may interrupt or even spoil their holidays. Some of the small tumours with less symptoms, and probably with better prognosis, might not be diagnosed in summer, and some of the tumours diagnosed in August or September may be those with a delayed diagnosis from the summer months, and thus, with a poorer prognosis.

Because of the large number of breast cancer patients in our study, even small survival differences reach statistical significance. In the case of breast cancer patients, the difference in the 5-year cumulative RSR between the month with the best prognosis and that with the worst was 4.8% units, and the corresponding figure for the colorectal cancer patients was 8.3% units. These figures can be interpreted in a following way: 1,182 patients died of the 2,657 breast cancer patients diagnosed in August during the study period 1956–1985 as only 316 deaths were expected, yielding 866 excess deaths among these patients. If the (stage adjusted) observed and expected 5-year cumulative survival rates of patients diagnosed in March were used for patients diagnosed in August, only 761 excess deaths would have occurred. The difference in the number of excess deaths was 105 or 12%. For colorectal cancer patients a similar calculation reveals 60 or 10% more excess deaths among those patients diagnosed in August as compared with those diagnosed in October.

It is likely that general factors associated with the health services and the health behaviour of women rather than biological factors cause these differences. This result should inspire clinicians to consider, if the quality of care in each individual clinic could be improved in summer.

### References

GALEA, M.H. & BLAMEY, R.W. (1991). Season of initial detection in breast cancer (letter). *Br. J. Cancer*, **63**, 157.

HAKULINEN, T. & ABEYWICKRAMA, K.H. (1985). A computer program package for relative survival analysis. *Comput. Programs Biomed.*, **19**, 197–207.

HAKULINEN, T. & TENKANEN, L. (1987). Regression analysis of relative survival rates. *Applied Statistics*, **36**, 309–317.

JOENSUU, H. & TOIKKANEN, S. (1991). Association between the month of diagnosis and prognosis in breast carcinoma. *Br. J. Cancer*, **64**, 753–756.

MASON, B.H., HOLDAWAY, I.M., STEWART, A.W., NEAVE, L.M. & KAY, R.G. (1990). Season of initial discovery of tumour as an independent variable predicting survival in breast cancer. *Br. J. Cancer*, **61**, 137–141.